# Report of Several Cases of Asiatic Cholera Successfully Treated by Chloroform

**Published:** 1849-08

**Authors:** Anthony Bournonville

**Affiliations:** Philadelphia


*Report of several cases of Asiatic Cholera successfully treated by Chloroform.* By Dr. Anthony Bournonville, Philadelphia.

Case 1. J——, living in the city, was seized during the night of the 14th of June, with vomiting, and purging of rice-water-like discharges, severe cramps in his stomach and calves of legs, tongue cold, pulse very small and feeble and slow, extreme prostration ; complained of excessive thirst and burning heat of his stomach ; skin presented a peculiar shrivelled appearance and doughy feel.

*Treatment.*—I administered at once chloroform *m.* vj., turpentine gtt. 40, in a glass of brandy and water, and repeated the dose in a half hour with happy effect; the vomiting and cramps ceased entirely after the second dose; sinapisms were applied to the calves of legs and stomach; he was well rubbed with cayenne pepper and brandy; the remainder of the treatment consisted of a mixture composed as follows, of which he took a tablespoonful every hour or two according to circumstances.

℞. Aquæ Camphorat. ℥iij.

Sol. Morph. Sulphat. ℥ss.

Spts. Lavend. Comp. ʒiij.

Syr. Sacchar. ℥ss.

M.

At the same time the patient took small pieces of ice, and very cold water in small quantities, as often as desired. As soon as the chloroform and turpentine were administered, the patient revived, not entirely however until the second dose was given; the heat of surface gradually returned; the pulse slowly increased in strength and frequency; the extreme prostration disappeared. I left the patient in a very comfortable situation an hour after first seen. The next day the patient expressed himself well, with the exception of being very weak; tonics were prescribed, and the patient was entirely well on the third day.

Case 2. George C——, undertaker, Vine and Fifth streets, was called upon to lay out for burial a patient who had died of Asiatic cholera. C. returned to his work after he had finished, took a glass of beer, and soon after was obliged to go to the privy, where he had a succession of copious watery evacuations. On passing through the yard to go into the dwelling house, he fell prostrate on the pavement, stiff, cold as ice, cold perspiration flowed freely from his body, (melting as it were) pulseless, skin shrivelled, extremities in less than half an hour were perfectly blue to his knees and elbows; tongue cold, breath cold, eyes sunken with livid circles about them, lips blue, excessive cramps in the stomach feeling like knots, nose contracted, ghastly appearance.

*Treatment.* Chloroform *m* vj. turpentine gtt. 40, were given in a glass of brandy and water, which caused immediate partial revival; the patient expressed himself much better; in half an hour the dose was repeated, which caused entire revival. Sinapisms were applied to the calves of the legs and stomach, was well rubbed with cayenne pepper and brandy, mustard and cayenne pepper, flowers of sulphur, heated bricks were applied to various parts of the body ; ordered afterwards the following mixture, of which he took a teaspoonful every half hour.

℞. Chloroform,* gtt.xxx.

Water, ℥ij.

Mix.

Saw the patient two hours after, pulse good but rather feeble, heat of surface gradually returning; no cramps, no nausea; extremities of hands and feet still blue; face presenting a natural appearance ; prescribed calomel, grs. v., inspissated ox gall, grs. x., made into four pills, of which he took two at once, and repeated two hours after, these produced good bilious evacuations; the rubbings were continued to hands and feet. The next day I found the patient much better, the blueness had entirely disappeared, the pulse stronger, some slight excitement present, some difficulty of breathing, slight congestion of the lungs. He was bled, and on the fourth day was entirely well with the exception of debility, for which quinine gr. ss. every half hour was given, with other tonic treatment. The patient is now well.

Case 3. W——, Callowhill above 13th, laboring under Diarrhœa of some days standing; was seized on the night of the 27th of June, at 11 o'clock, with vomiting, and purging of rice water-like discharges; excessive cramp in stomach. A physician in the neighborhood was called in, who prescribed the remedies proper for symptoms then presenting themselves; the vomiting and purging continued at intervals during the night; cramps in the legs ensued. I was sent for next morning, and arrived there at 9½ o'clock, not knowing that a physician was in attendance, and not thinking to ask in the hurry of the moment, and having attended her family for 18 years, I prescribed. I found the patient cold, pulseless, cramped in the stomach and legs, vomiting and purging incessantly ; tongue cold, breath cold, blue and shrivelled appearance of arms and legs, contracted features, sepulchral voice, burning heat at stomach :—in fact, presenting to my view one of

*The Chloroform mixture is prepared some few days beforehand, as follows :—thirty drops are dropped into a two ounce vial, which is afterwards filled with warm water, and tightly corked—this is allowed to cool; before giving the necessary dose be careful to shake well the mixture.

the most malignant cases of Asiatic cholera, in a collapsed state, that I have ever seen; the cramps in the legs were most violent, causing the muscles to contract violently and feel as hard as a board. Treatment :—I administered (without any hope of success) chloroform *m.* vi. turpentine gtt. 40 in a glass of brandy and water, which caused the vomiting and purging to cease at once; the cramps were not relieved; sinapisms—rubbings were ordered as in other cases above mentioned. She refused by signs to take any more of the remedy; her powers of nature being exhausted, she died a few hours after.

Case 4. Mrs. R. ——, Front St. opposite Peg, keeper of a vegetable and meat shop, living in a dirty house, kept constantly damp by frequent drenching of floors with water, and the atmosphere rendered foul by exhalations arising from decayed vegetable matters, was taken on the 26th June with diarrhœa, which she allowed to run on for a few days before she sought medical advice. On the 28th I was called in a hurry to see Mrs. R. whom I found laboring under severe diarrhœa with constant retchings, but no vomiting; she complained of rumbling pains in her stomach and bowels; pulse natural, temperature of body perfectly natural in every respect. The camphorated anodyne mixture, spoken of before, was given in tablespoonful doses every hour—the abdomen to be rubbed with a liniment composed of

| | | |
|---|---|---|
| ℞. | Gum. Camphoræ | ʒi. |
| | Ol. Olivæ | ℥i. |
| | Tr. Opii. | ℥ij. |
| | Aquæ Ammoniæ fort. | ℥ss. |
| | M. | |

The remedies gave her no relief; the diarrhœa continued to a greater degree; the patient vomited the medicine; ordered injections of starch and laudanum, and prescribed one of the following pills to be given every two hours:

| | | |
|---|---|---|
| ℞. | Hydrarg. C. Mitis. | gr. viii. |
| | Pulv. Opii. | gr. iv. |
| | G. Camphor. | grs viii. |
| | M. Ft. pil. No. 8. | |
| | S. One every two hours. | |

No relief was experienced from these; vomiting and purging of rice water fluid ensued at short intervals; cramps in stomach and legs; next morning I found her collapsed, pulseless; legs and arms blue to knees and elbows; skin shrivelled, doughy and cold as ice; respiration slow and sighing; breath cold; tongue cold; sepulchral voice; terrible burning heat in stomach.

Chloroform *m.* vj. turpentine gtt. 40, in a glass of brandy and water were given at once, and immediately rejected from the stomach; then gave her a tablespoonful of the following mixture every half hour for two hours, then every hour.

Chloroform, gtt. xxx.
Aquæ Fontan. ℥ii.
M.

This had the effect of checking the vomiting and cramps—it also allayed the diarrhœa; the cold state continued; usual rubbings with cayenne and brandy; mustard and cayenne, flowers of sulphur, combined with sinapisms to legs and stomach; warmth by means of heated bricks moistened with vinegar were resorted to without benefit. For the burning heat of stomach small pieces of ice were given at short intervals: no nourishment of any kind allowed. The chloroform was grateful to her stomach, and afforded her immediate partial relief. The chloroform was continued as above for two days, the diarrhœa, vomiting and cramps, in the meanwhile, ceased entirely, but still the patient remained in a collapsed state. At the expiration of this time, calomel grs. v. inspissated ox gall grs. x., made into four pills were ordered, of which she took two at once, and repeated two hours after. These produced good bilious stools; she expressed herself much better, but was still in a collapsed state. The next day the diarrhœa recurred, with pains in her stomach; the chloroform mixture was again resorted to in tablespoon doses every hour. The pains ceased, diarrhœa still continued, for which injections of pulv. opii. grs. ii. plumb. acet. grs. x. were ordered, and the chloroform mixture continued; the diarrhœa ceased; patient expressed herself much better after each dose of the chloroform. She remained in a collapsed state the greater part of five days; at the end of the fifth day violent reaction ensued, the face lost that ghastly appearance and blueish hue; became flushed and full; the eyes became prominent and bright; pulse increased in fulness and frequency. The chloroform mixture was now discontinued, and in its place ordered liq. anod. Hoffman ℥ss., spts. ammoniæ aromat. ʒi.

twenty-five drops to be given every two hours in sweetened water. The next day patient relapsed; pains recurred; diarrhœa of a light chocolate color continued; the chloroform was again resorted to in the usual dose; injections of acetate of lead and opium in same proportions as above were given. The pains were relieved; the diarrhœa ceased; reaction recurred to a proper extent; the extremities became warm; the blue and shrivelled appearance disappeared; the pulse became fuller, quite regular, but very feeble; the face resumed a natural appearance and expression; the respiration became more regular; the chloroform mixture was now given in more moderate doses (teaspoonful every hour) and ordered calomel gr. $\frac{1}{4}$, opium gr. $\frac{1}{4}$, every two hours for sixteen hours. The bowels became costive, relieved by ol. ricini ℥ss. Ever since she has been taking quiniæ sulphat gr. ss. every two hours, combined with other tonic treatment and is now rapidly recovering.

[It may be well to observe that this remarkable case was visited several times daily by my son, Dr. Aug. C. Bournonville, as well as by many of the neighbors, who all manifested a lively interest for the recovery of Mrs. R——. The patient is now (July 20) perfectly well.]

Case 5. R——, Chestnut and Second sts., was attacked with slight diarrhœa, with rumbling pains in stomach, for which I prescribed the camphorated anodyne mixture, sinapisms, frictions, &c. as in other cases. These gave no relief; the next morning she was seized with vomiting and purging of rice water fluid; cramps in stomach; no cramps in legs; pulse scarcely distinguishable; temperature of body reduced; cold tongue; slight blueness of extremities; respiration slow and sighing. I gave her at once a tablespoonful of the chloroform mixture (gtt. xxx. to ℥ii.) and repeated in half an hour; this caused the vomiting and cramps to cease at once; the patient revived and expressed herself much better; the pulse increased in frequency and strength; the surface of the body became warmer; the blueness of extremities gradually disappeared; the chloroform was continued for two days together with anodyne injections; the diarrhœa and vomiting ceased; at the expiration of this time the chloroform was discontinued, and in its place cal. gr. v. inspis., ox gall grs. x., made into four pills; two to be taken at once and repeated two hours after, were ordered; no action of the bowels ensued; a dose of castor oil with laudanum was given; diarrhœa and pains slightly recurred; gentle anodyne mixture and injections were prescribed; these gave no relief; the pains increased, and retching ensued. The chloroform mixture was administered with decided relief, and continued in teaspoonful doses every hour; the pains and diarrhœa ceased entirely. The bowels became costive, remedied by injection of salt and water; the evacuations became regular, and of good color; the chloroform was discontinued; quinine and other tonic remedies prescribed, and the patient is now well.

Case 6. U——, N. Tenth above Race st., was seized early in the morning with vomiting and purging; with cramps in the stomach and legs; great prostration; temperature of body reduced; the evacuations were copious, and of a very light chocolate color; pulse slow and feeble. The chloroform mixture was again used in this case in tablespoonful doses, repeated in half an hour. The symptoms were relieved at once; sinapisms, &c., were used as in other cases. The mixture was continued in teaspoonful doses every hour; the next day the patient had entirely recovered from all the symptoms with the exception of the debility, for which quinine and other tonic remedies were prescribed. In a few days the patient had entirely recovered.

Case 7.—G——, living in the same house (Callowhill above 13th,) in which W——, case 3, died, was attacked with diarrhœa and rumbling pains in his stomach and bowels, and considerable feeling of weakness. For these he resorted to various remedies suggested by friends. A few days after, I was called in a hurry to see Mr. G.; found him vomiting and purging; the dejections from the stomach were slimy, those from the bowels rice water-like; complained of severe cramp in stomach and legs; very much prostrated; tongue cooler than natural; extremities slightly blue; pulse very small and feeble; respiration oppressed; surface of body cooler than natural. After the vomiting ceased there were constant retchings. The chloroform mixture was administered in the same dose and with the same results as mentioned in other cases; the mixture was continued for two hours; frictions, sinapisms and other measures were resorted to as in other cases above mentioned. Pills of camphor, opium, and capsicum were given every other hour alternating with the camphorated anodyne mixture mentioned before. Slight diarrhœa continued, with much pain, for which the chloroform was ordered. The second day after, he was well, with the exception of debility, for which tonics were prescribed.

Case 8. C——, N. 3d above Tamany st., was seized in the street with cramps in stomach and calves of legs, with constant vomiting and purging; pulse very small and feeble; skin cooler than natural; patient very much prostrated. He was taken into an apothecary's shop, where laudanum was administered. The vomiting continued; his friends desired that he should be taken to the Hospital in the immediate neighborhood; the patient however, desired that I should be sent for; I arrived as soon as possible, and found him as above described. The chloroform mixture was given at once in tablespoonful doses, and repeated in half an hour with decided relief. Frictions, &c. were made use of; the mixture was continued in teaspoonful doses every hour after. The next day I found the patient doing very well; tonics were prescribed, and patient in a day or two was entirely well.

*Cases treated by Aug. C. Bournonville.*

Case 1. Henry R——, constable, aged 42. Habits intemperate; was laboring under diarrhœa for several days; attended a 4th of July dinner; was seized on the 5th of July at 9½ in the morning with vomiting and purging of rice water fluid; cramps in stomach, legs, and arms; body perfectly cold—pulseless; extremities blue and shrivelled; blueness under eyes; lips blue. I was sent for, but was not at home at the time. Drs. Sargent and Wilson of the Cherry street Hospital were called upon; they visited the patient at his house, and found him in the condition above described; injections of tr. opii ʒi. were given; mustard sinapisms were applied to the calves of legs and stomach; external warmth by means of heated bricks was resorted to; ferri sulphat. gr. i., quiniæ sulphat. i. gr. every hour were ordered to be given. Drs. S. and W. left him at the end of an hour worse than when first seen; tongue cold, breath cold,—in fact perfectly collapsed. As soon as I returned home I called upon R., found that he had been prescribed for, and retired. I was sent for at 3 o'clock with a message saying that the patient was dying. At this time found the patient in violent cramps of stomach and calves of legs and fingers; breath cold, tongue cold, hands and feet blue; complained of excessive burning heat of stomach; respiration oppressed and sighing; action of heat very feeble.

Chloroform *m.* vi. turpentine gtt. 40, in brandy and water, were given at once and repeated in half an hour; sinapisms to legs, arms and stomach; frictions with cayenne and brandy. The chloroform mixture (gtt. xxx. to aq. ℥ii.) was then given in teaspoonful doses every half hour. I remained with him an hour and a-half, at the end of which time the patient expressed himself better; the cramps in stomach, and nausea had left him; the cramps in legs had moderated; the sinapisms to the calves of legs had acted slightly; the respiration was easier; the pulse was scarcely distinguishable. Ice and brandy were given at intervals, frictions continued.

At 5½. Patient expressed himself much better. Gave calomel gs. v., ox gall grs. x., made into four pills, two at once, and repeated in two hours.

At 6½. Found him worse; pulseless, uneasy, action of heart excessively feeble; continued chloroform mixture in tablespoonful doses, every half-hour; brandy and ice at short intervals.

At 7½. Patient, to use his own words, expressed himself "much better, with exception of cramps in fingers;" head, neck, legs and stomach warm; pulseless; respiration easy; arms perfectly cold and blue.

At 9½. Found him in frightful cramps of the whole body; legs drawn up, respiration gasping; patient continued thus until he died at 11¾ P. M.

Case 2. Mrs. R——, aged 34—U. S.—temperate—married; was seized at 6½ o'clock A. M. with vomiting of slimy matter, purging of rice water fluid, cramps in stomach and legs, and one arm; constant disposition to vomit afterwards; very much prostrated; pulse small and feeble; action of heart regular; respiration rather slower than natural; temperature of body and tongue reduced.

I gave chloroform mixture, tablespoonful at once, and repeated in half an hour; this dispelled the nausea at once; the cramps were moderated; the patient expressed herself much better, and when asked remarked that as soon as she took the mixture, she experienced a cordial heating sensation in the stomach, which diffused itself throughout the body; discontinued the mixture, and gave the anodyne camphorated mixture mentioned heretofore ; tablespoonful every hour ; saw the patient two hours after, found her much better ; warm perspiration, very feeble, respiration easier, pulse good, bowels opened twice, and evacuations of a light chocolate color ; no cramps since ; continued the camphorated anodyne mixture.

Saw her in the afternoon ; slight nausea, occasional slight cramps in her legs ; sense of coldness ; pulse good ; chloroform mixture was given in teaspoonful doses every hour ; continued the other mixture ; next morning found her much better ; no cramps, heat of surface natural ; slept well during the night ; pulse regular but very feeble ; quiniæ gr. ss. every hour and other tonic remedies ordered. Ten days after, entirely well.

Case 3. Mrs. R——, aged 65, U. S. ; temperate ; shop-keeper, mother of cases first and second ; was taken with diarrhœa of a light color and watery ; pulse feeble ; prescribed camphor, gr. i., opium, gr. ss., acetate of lead gr. i., every hour ; diarrhœa ceased in four hours ; continued with the camphorated mixture in teaspoonful doses every hour. At twelve of same night, patient was seized with vomiting and purging of rice water fluid, cramps in stomach and legs, body perfectly cold, pulse scarcely perceptible, fingers shrivelled. The chloroform mixture was given in tablespoonful doses, every half-hour ; sinapisms were applied to legs and stomach. In half an hour expressed herself much better ; in less than half an hour the nausea disappeared, the heat of surface returned, the cramps were relieved, the pulse increased in frequency and fulness, body bathed with warm perspiration. I may add, however, that the first dose was rejected with other fluids from the stomach ; the dose was given as soon as the vomiting ceased, and continued every half-hour for two hours. The chloroform was then discontinued, and the camphorated mixture given instead, tablespoonful every hour.

In the afternoon of same day, prescribed calomel, gr. ¼, op. gr. ss., pulv. rad. ipecac. gr. ¼, every two hours ; this was rejected ; slight cramps in stomach recurred ; slight nausea ; discontinued powders and gave chloroform mixture, teaspoonful every half-hour at first, and afterwards every hour, alternating with the camphorated mixture ; next day prescribed quinine gr. ss. every hour, and other tonic remedies, and is now slowly recovering.

